# Evolutionary response to global change: Climate and land use interact to shape color polymorphism in a woodland salamander

**DOI:** 10.1002/ece3.3118

**Published:** 2017-06-12

**Authors:** Bradley J. Cosentino, Jean‐David Moore, Nancy E. Karraker, Martin Ouellet, James P. Gibbs

**Affiliations:** ^1^ Department of Biology Hobart and William Smith Colleges Geneva NY USA; ^2^ Direction de la recherche forestière Ministère des Forêts, de la Faune et des Parcs Québec City QC Canada; ^3^ Department of Natural Resources Science University of Rhode Island Kingston RI USA; ^4^ Amphibia‐Nature Montréal QC Canada; ^5^ College of Environmental Science and Forestry State University of New York Syracuse NY USA

**Keywords:** adaptive evolution, climate change, eastern red‐backed salamander, forest ecology, landscape change, morphology, natural selection, *Plethodon cinereus*

## Abstract

Evolutionary change has been demonstrated to occur rapidly in human‐modified systems, yet understanding how multiple components of global change interact to affect adaptive evolution remains a critical knowledge gap. Climate change is predicted to impose directional selection on traits to reduce thermal stress, but the strength of directional selection may be mediated by changes in the thermal environment driven by land use. We examined how regional climatic conditions and land use interact to affect genetically based color polymorphism in the eastern red‐backed salamander (*Plethodon cinereus*). *P. cinereus* is a woodland salamander with two primary discrete color morphs (striped, unstriped) that have been associated with macroclimatic conditions. Striped individuals are most common in colder regions, but morph frequencies can be variable within climate zones. We used path analysis to analyze morph frequencies among 238,591 individual salamanders across 1,170 sites in North America. Frequency of striped individuals was positively related to forest cover in populations occurring in warmer regions (>7°C annually), a relationship that was weak to nonexistent in populations located in colder regions (≤7°C annually). Our results suggest that directional selection imposed by climate warming at a regional scale may be amplified by forest loss and suppressed by forest persistence, with a mediating effect of land use that varies geographically. Our work highlights how the complex interaction of selection pressures imposed by different components of global change may lead to divergent evolutionary trajectories among populations.

## 1 INTRODUCTION

Human impacts on biodiversity are often examined in isolation, but components of global change can act simultaneously on populations in an additive or synergistic fashion (Brook, Sodhi, & Bradshaw, 2008; Mantyka‐Pringle, Martin, & Rhodes, 2012). For example, habitat loss and fragmentation can amplify the ecological effects of climate change by creating extreme microclimates, limiting the ability of species to move, or altering species interactions (Brook et al., 2008; Opdam & Wascher, 2004; Urban, Zarnetske, & Skelly, 2013). Studies are revealing how climate and land use interact to affect species distributions and habitat suitability (e.g., Brodie, 2016; Forister et al., 2010; Mantyka‐Pringle, Martin, Moffatt, Link, & Rhodes, 2014; Nowacki & Abrams, 2014; Nowakowski et al., 2017), but little is known about how these factors together affect adaptive evolution, a critical process for facilitating response of dispersal‐limited species to environmental change (Carlson, Cunningham, & Westley, 2014).

Climate change is expected to cause directional selection for traits that reduce physiological stress in response to increasing temperature and drought (Hoffmann & Sgrò, 2011). However, landscape change also plays an important role in shaping temperature and moisture conditions experienced by organisms. In forested regions, loss and fragmentation of forest increases solar exposure and outgoing radiation, creating warm conditions at the soil surface (Chen et al., 1999; Matlack, 1993; Murcia, 1995). Soil temperatures are on average 7–9°C warmer at forest edges than interior, and these thermal changes are consistent across forest types and latitudes (Tuff, Tuff, & Davies, 2016). Furthermore, temperature and moisture conditions at the forest floor are not independent. Forest fragments with warm temperatures tend to be desiccating environments due to high evapotranspiration rates and consequently low soil moisture (Chen et al., 1999; Laurance, 2004; Murcia, 1995). Temperature differences between forest fragments and deforested matrix habitat also generates a “vegetation breeze” that leads to forest fragments receiving less moisture from rainfall than the adjacent matrix (Cochrane & Laurance, 2008; Garcia‐Carreras & Parker, 2011). Collectively this suggests that forest cover may play an important role in mediating directional selection imposed by changing macroclimatic conditions. Directional selection caused by climate change may be amplified by forest loss and dampened in areas experiencing forest regeneration. Relating geographic variation in genetically based traits to climate and land use can provide much needed insight into how climate change and landscape modification interact to drive evolutionary trajectories.

Eastern red‐backed salamanders (*Plethodon cinereus*) in forests of the eastern United States and southeastern Canada present a valuable opportunity to explore evolutionary response to the combined effects of climate and land use. *Plethodon cinereus* is a terrestrial, lungless salamander that relies entirely on respiration across permeable skin, and the skin must remain moist for adequate gas exchange (Spotila, 1972). Warm, dry conditions reduce foraging and reproductive activity and increase metabolic costs to individuals (Homyack, Haas, & Hopkins, 2011; Jaeger, 1980), so temperature and moisture are key constraints on fitness. Moreover, *P. cinereus* has limited dispersal ability, and therefore, the potential to respond to changing climatic conditions will likely depend in part on evolution within populations.

*Plethodon cinereus* has two primary discrete color morphs (striped and unstriped dorsal surface, Moore & Ouellet, 2014) that are genetically based (Highton, 1959, 1975), and morph frequencies have been linked to geographic variation in macroclimatic conditions. Previous studies indicated unstriped individuals are more closely associated with warmer climates than striped individuals (Fisher‐Reid & Wiens, 2015; Gibbs & Karraker, 2006; Lotter & Scott, 1977; Moreno, 1989), and the unstriped morph has also been linked to drier conditions than the striped morph (Anthony & Pfingsten, 2013; Fisher‐Reid, Engstrom, Kuczynski, Stephens, & Wiens, 2013). However, a recent rangewide analysis indicated morph frequencies are spatially variable within climate zones (Moore & Ouellet, 2015), and physiological studies have failed to find consistent morph‐specific moisture tolerances (Heatwole & Lim, 1961; Smith, Johnson, & Smith, 2015).

Temperature and moisture conditions experienced by salamanders at the forest floor depend in part on the amount of solar penetration through the forest canopy (Peterman & Semlitsch, 2013). Thus, forest cover may play an important role in mediating directional selection imposed by macroclimatic conditions on *P. cinereus* color morphology, resulting in spatial variation in morph frequencies in regions with consistent climate but variable forest extent. Moreover, the degree to which macroclimatic effects are mediated by forest cover may itself depend on macroclimatic conditions. For example, temperature increases driven by forest loss in cold regions may be insufficient to reach the range of thermal conditions tolerated by unstriped individuals, and limited polymorphism may constrain evolutionary response to land use changes in some regions. Variation in the mediating effect of land use on climate can be detected by examining an interaction between forest cover and climate variables on morph frequencies.

We integrated two compilations of color morph frequencies in *P. cinereus* reported since the 19th century (Gibbs & Karraker, 2006;  Moore & Ouellet, 2015) with new data to test whether forest cover acts in an additive or interactive fashion with ambient temperature and precipitation to shape spatial variation in color morph frequencies. We also tested for temporal variation in morph frequencies that may be associated with changing macroclimatic conditions across the geographic range of *P. cinereus* (e.g., climate warming). We used path analysis to deduce the effects of ambient temperature, precipitation, forest cover, and sampling year on morph frequencies in populations while accounting for associations among the explanatory variables.

## 2 MATERIALS AND METHODS

### 2.1 Color morph frequencies

We combined two data compilations including published and unpublished reports of morph frequencies from studies conducted between 1880 and 2013 (Gibbs & Karraker, 2006; Moore & Ouellet, 2015), and we added unpublished data on morph frequencies assembled by the authors for 2013–2015. Variables from the two compilations included sampling year, geographic coordinates (latitude, longitude), total number of individuals sampled, number of striped and unstriped individuals, and the data source (published literature or contact information for unpublished data). We used the median year as sampling year when data were collected across multiple years, and erythristic individuals (<1% of individuals) were classified as striped in the original compilations (Gibbs & Karraker, 2006; Moore & Ouellet, 2015). The combined dataset consisted of 1,472 unique entries (242,704 individuals) for data collected between 1880 and 2015.

### 2.2 Climate and land use variables

The resolution of geographic coordinates for sites varied greatly among studies and ranged from virtually exact locations to town‐ or county‐level coordinates. To accommodate uncertainty about site locations, we used Geospatial Modeling Environment (Beyer, 2014) to create a standardized grid of 50 × 50 km landscape blocks that included all study sites (Fig. S1). All sites within the same landscape block were assigned the same values for climate and land use variables.

We used the Climatic Research Unit time series version 3.23 (Harris & Jones, 2015) to quantify mean annual temperature (°C) and mean annual precipitation (mm) from 1901 to 2014 for each landscape block. Mean annual temperature and precipitation were employed to characterize spatial variation in macroclimatic conditions among sites. We considered using climate estimates that were aligned with the timing of sampling (i.e., year‐ or month‐specific estimates) because some studies have indicated seasonal variation in the activity of color morphs (e.g., Anthony, Venesky, & Hickerson, 2008; Lotter & Scott, 1977; Test, 1955). However, we used mean annual estimates because morph frequency data for many studies were carried out over variable timeframes (including multiple seasons), and information on seasonality of data collection was not always available. Moreover, climate data specific to the sampling time represents stochastic variation to some degree, whereas our focus was on deterministic, evolutionary response to climate over multiple generations (minimum 3‐year generation time; Sayler, 1966).

We used a 250‐m resolution dataset representing land cover for North America in 2010 (Commission for Environmental Cooperation, 2013) to quantify the proportion of forested habitat within each landscape block. We combined the following land use classifications to index overall forest cover: (1) temperate or subpolar needleleaf evergreen forest, (2) temperate or subpolar broadleaf deciduous forest, and (3) mixed forest.

### 2.3 Statistical analyses

We used path analysis (Grace, 2006; Kline, 2015) to examine how proportion of striped individuals was related to temperature, precipitation, forest cover, and sampling year. Path analysis was favored over alternative approaches (e.g., multiple regression) because exploratory analysis revealed geographic biases in sampling over time (e.g., tendency for cool, forested regions to be sampled late in the sampling period). Path analysis allowed us to examine a direct effect of sampling year on morph frequencies while controlling for associations between sampling year and other explanatory variables.

We fit two models to examine hypothesized direct effects (i.e., regressions) of sampling year as well as additive and interactive effects of climate variables and forest cover on proportion of striped individuals. Each model included direct effects of temperature, precipitation, forest, and sampling year on proportion striped. One model included an interaction effect between temperature and forest cover on proportion striped, and the second model included an interaction effect between precipitation and forest cover on proportion striped. Interaction effects were specified by including a direct effect of product terms (temperature × forest or precipitation × forest) on proportion striped. Temperature, precipitation, and forest cover were centered to minimize the covariance between each variable and the product term (Kline, 2015). We included covariance terms between variables in our models to represent correlations generated by factors that were not modeled (Grace, 2006). Both models included covariance terms between temperature and forest cover, precipitation and forest cover, and temperature and precipitation. We also included covariance terms between the product term representing the interaction effect and (1) sampling year, and (2) the climate variable not modeled as an interaction effect (e.g., a covariance term was included between precipitation and the temperature × forest product term). We illustrated the strength of interaction effects using a composite variable including the main effects and product term (Grace, 2006).

**Figure 1:**
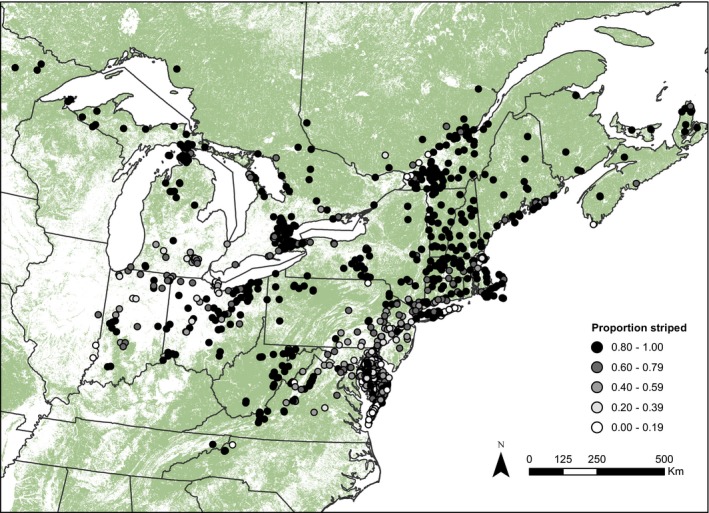
Map of forest cover (green) and study sites (circles) for eastern red‐backed salamanders (*Plethodon cinereus*) sampled from 1880 to 2015 in North America. Symbol fill for study sites represents observed proportion of striped *P. cinereus*

Before fitting each path model, we excluded all sites that had <20 individuals to increase precision (reduce contribution of sampling error) of estimates of proportion striped, resulting in 1,170 sites (including 238,591 individuals) for analysis (Figure 1). We applied a logit transformation to proportion striped (Warton & Hui, 2011), and we rescaled variables to be similar in magnitude (Rosseel, 2012). The *lavaan* package (Rosseel, 2012) in R (R Core Team 2016) was used to fit the models, and we employed the *lavaan.survey* package (Oberski, 2014) to estimate robust standard errors to account for the clustering of sites within landscape blocks. We compared the importance of the effects of explanatory variables on proportion striped by comparing the strength and significance of standardized coefficients for each pathway. Because standardized coefficients can depend on the variance of each variable, we also reported unstandardized coefficients (Grace & Bollen, 2005). We used χ^2^ and root mean square error of approximation (RMSEA) as model fit indices. Good model fit is indicated by *p > *.05 for the χ^2^ test and RMSEA <0.05 (Kline, 2015).

After examining the path models including interaction effects, we fit a reduced model that eliminated path coefficients that were not significant in the original models. We then compared model fit tests and the coefficient of determination (*R* ^2^) between the original and reduced models to examine if the reduced model adequately fit the data. We performed additional analyses to ensure that the results of our final path model were robust. First, we examined sensitivity of the results to criteria about minimum sample size and size of landscape blocks. We re‐analyzed the path model using all combinations of four minimum sample sizes (20, 30, 40, or 50 individuals) and three landscape block sizes (10 × 10, 50 × 50, and 100 × 100 km). Second, we examined whether our results were biased by spatial overrepresentation of sites (e.g., high density of sites in Delmarva Peninsula; Figure 1). We re‐analyzed the path model using one randomly selected site from each 50 × 50 km landscape block. This procedure was repeated 1,000 times enabling generation of 95% confidence intervals for estimates of standardized coefficients from the 1,000 iterations.

## 3 RESULTS

**Figure 2:**
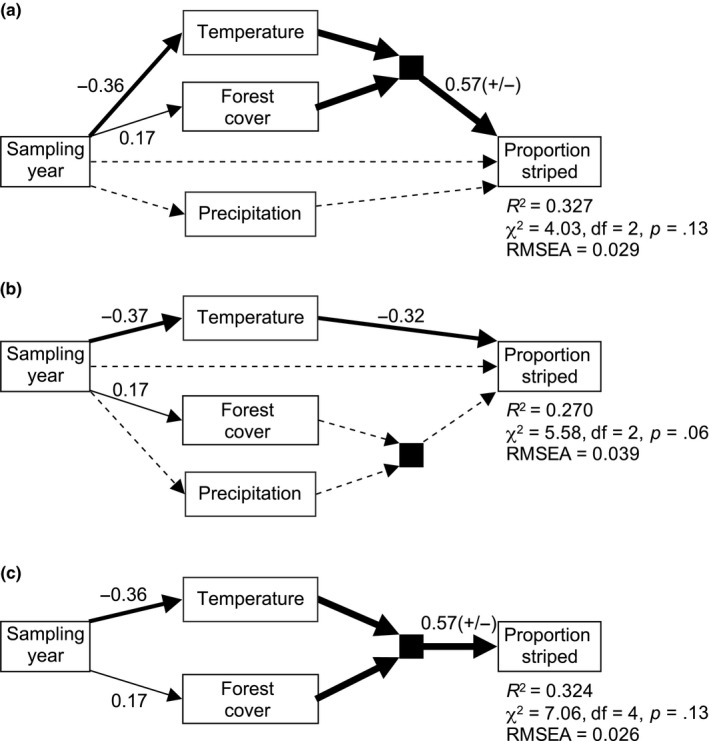
Path analyses of eastern red‐backed salamander (*Plethodon cinereus*) morph frequencies at 1,170 sites in Canada and the United States. Models included two full models with either (a) temperature × forest or (b) precipitation × forest interaction effects on striped frequency and (c) a reduced model with significant effects only. Only relationships hypothesized to be causal are shown. Solid lines represent significant pathways at *p *≤ .05, and dashed lines represent coefficients that were not significant. Standardized coefficients are shown for each pathway significant at *p *≤ .05, and the absolute magnitude of interaction effects are represented by a composite variable including the main effects and a product term (filled square). Model fit indices are shown for each model

**Figure 3:**
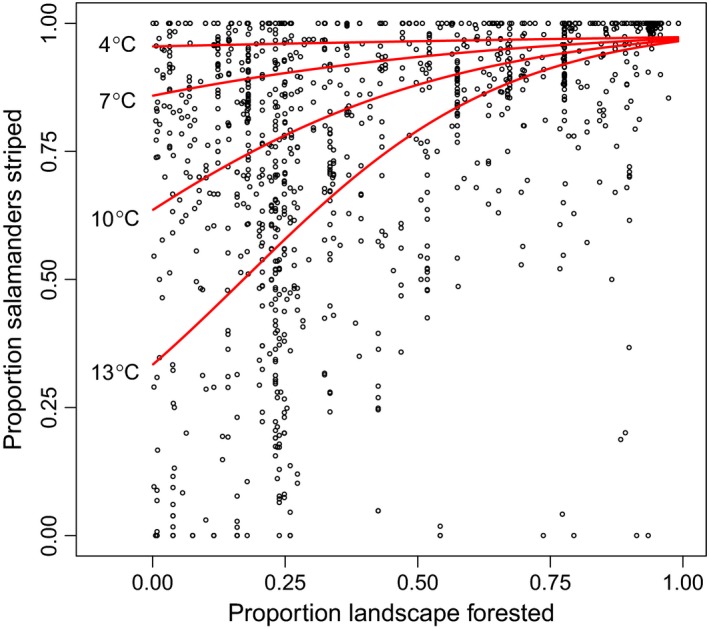
Relationship between the proportion of striped eastern red‐backed salamanders (*Plethodon cinereus*) and proportion of landscape forested at 1,170 sites in Canada and the United States. Best‐fit lines represent effects of forest cover on proportion striped at varying mean annual temperature based on regression coefficients from the reduced path analysis model with a temperature × forest interaction effect on striped frequency (Table 1 and Figure 2c)

Our models showed that *P. cinereus* morph frequencies were significantly related to temperature and forest cover but not to precipitation or sampling year (Figure 2 and Table 1). Moreover, there was a significant interaction effect between temperature and forest cover on proportion of striped individuals (Figure 2 and Table 1). Proportion striped was positively related to forest cover at warm temperatures, but the relationship between proportion striped and forest cover steadily weakened as temperature decreased regionally (Figure 3). There was no significant interaction between precipitation and forest cover, nor was there evidence for an additive effect of precipitation on proportion striped (Figure 2 and Table 1). Temperature was negatively related to sampling year, and forest cover was positively related to sampling year; however, there was no evidence for a direct effect of sampling year on proportion striped (Figure 2 and Table 1). Model fit was good for each of our path models, and a reduced model that eliminated nonsignificant path coefficients had a virtually identical *R* ^2^ value (32.4%) compared to the more complex model with a temperature × forest interaction effect and direct effects of precipitation and sampling year (*R* ^2^ = 32.7%; Figure 2). Our results were not sensitive to choice of sample size for site inclusion, spatial scale at which temperature and forest cover were quantified, or spatial variation in the density of sites (Tables S1 and S2).

**Table 1:** Unstandardized and standardized coefficients for path analyses of eastern red‐backed salamander (*Plethodon cinereus*) morph frequencies. Variables included striped frequency (S), temperature (T), precipitation (P), forest cover (F), sampling year (Y) and temperature × forest (TF) and precipitation × forest (PF) interaction terms. Models include two full models with either TF or PF interaction effects on striped frequency and a reduced model with significant path coefficients only

Model	Relationship	Variables	Unstd. coef.	SE	*p*	Std. coef.
T × F Interaction	Regressions	S ← T	−0.024	0.004	<.001	−0.343
		S ← F	0.272	0.037	<.001	0.349
		S ← T × F	0.036	0.012	.002	0.141
		S ← P	−0.007	0.008	.399	−0.042
		S ← Y	−0.030	0.029	.308	−0.032
		T ← Y	−4.711	0.935	<.001	−0.362
		F ← Y	0.203	0.064	.001	0.171
		P ← Y	0.237	0.359	.509	0.043
	Covariances	T, F	−0.211	0.068	.002	−0.253
		T, P	1.166	0.264	<.001	0.299
		P, F	0.067	0.030	.025	0.178
		Y, TF	−0.001	0.012	.913	−0.006
		P, TF	−0.364	0.080	<.001	−0.316
P × F Interaction	Regressions	S ← P	−0.014	0.008	.080	−0.088
		S ← F	0.262	0.041	<.001	0.346
		S ← P × F	0.036	0.026	.179	0.063
		S ← T	−0.021	0.004	<.001	−0.295
		S ← Y	−0.012	0.029	.690	−0.013
		T ← Y	−4.711	0.935	<.001	−0.368
		F ← Y	0.203	0.064	.001	0.171
		P ← Y	0.237	0.359	.509	0.043
	Covariances	T, F	−0.191	0.061	.002	−0.233
		T, P	0.826	0.308	.007	0.216
		P, F	0.091	0.034	.007	0.241
		Y, PF	0.003	0.005	.464	0.037
		T, PF	−0.255	0.069	<.001	−0.231
Reduced	Regressions	S ← T	−0.025	0.004	<.001	−0.347
		S ← F	0.261	0.035	<.001	0.335
		S ← T × F	0.039	0.011	<.001	0.152
		T ← Y	−4.711	0.935	<.001	−0.362
		F ← Y	0.203	0.064	.001	0.171
	Covariances	T, F	−0.211	0.068	.002	−0.253

## 4 DISCUSSION

Climate warming is predicted to cause directional selection for traits that reduce thermal stress (Hoffmann & Sgrò, 2011), but our results indicate that land use may be an important component of global change that complicates this prediction. We showed that ambient temperature and forest cover can interact to affect the spatial distribution of heritable traits. The proportion of striped *P. cinereus* in populations was greatest at cool, northern latitudes and in warm regions with high forest cover. Conversely, proportion striped was lowest in warm regions where urbanization and agriculture have led to extensive forest loss, particularly in the midwestern and mid‐Atlantic United States (Figure 1). The degree of variance in morph frequencies explained together by temperature and forest cover was surprisingly high (*R* ^2^ = 32.4%) given all the sources of heterogeneity in the dataset, including the large geographic scale of the analysis, variation in observers and sampling methods, spatial biases in sampling year, precision of geographic coordinates, and changing climate and land use patterns across the region. Overall, our results suggest that landscape change has the potential to amplify or dampen directional selection imposed by climate change.

We hypothesize that the positive relationship between proportion striped and forest cover at warm sites is due to the mediating effect of forest cover on microclimatic conditions experienced by salamanders at the forest floor. Landscapes with moderate forest loss have greater edge habitat compared to landscapes where forests are still intact (Fahrig, 2003). Edge effects associated with forest loss and fragmentation are known to create warm conditions at the forest floor due to sunlight and wind penetrance (Chen et al., 1999; Gehlhausen, Schwartz, & Augspurger, 2000; Matlack, 1993). Although there was no association between morph frequencies and annual precipitation levels, temperature and moisture at the forest floor may together affect morph‐specific fitness because of the tendency for warm conditions to create a desiccating environment with low moisture levels (Tuff, Tuff, & Davies, 2016). It is possible that the fitness of striped individuals is greater than unstriped individuals in cool, wet microclimates in landscapes with high forest cover, whereas fitness of unstriped individuals approaches or exceeds that of striped individuals in forest fragments with warm, dry microclimates. We suggest that variation in land use practices that affect forest cover may partly explain why morph frequencies are spatially variable even in regions with similar annual temperatures (Moore & Ouellet, 2015).

There are multiple potential explanations for why the mediating effect of forest cover was strongest at warm sites and virtually nonexistent at the coldest sites as indicated by the interaction effect between forest cover and temperature. First, the degree of warming caused by low forest cover at cold sites may be insufficient to overcome the negative effect of cold ambient temperature on fitness of unstriped individuals. Unstriped individuals tended to be less common at cool than warm sites, which may be due to cool conditions increasing physiological stress for the unstriped morph (Lotter & Scott, 1977; Moreno, 1989). However, populations with high frequencies of unstriped individuals do occur at cold, northern sites (Moore & Ouellet, 2015; Figure 1), and it is possible that the unstriped morph has become locally adapted to cold conditions in some populations. Experimental data on differential fitness between morphs at varying ambient temperature and forest cover are needed to provide mechanistic insight into how temperature and forest cover interact to shape thermal selection. Moreover, comparative studies are needed to identify behavioral and physiological traits linked to color morph that may be targets of thermal selection. Previous studies have compared many traits between color morphs, including metabolic rates (Moreno, 1989; Petruzzi, Niewiarowski, & Moore, 2006), dehydration/rehydration rates (Heatwole & Lim, 1961; Smith, Johnson, & Smith, 2015), stress physiology (Davis & Milanovich, 2010), growth rates and survival (Muñoz, Hesed, Grant, & Miller, 2016), movement behavior (Cosentino & Droney, 2016), and seasonal activity (e.g., Anthony et al., 2008; Lotter & Scott, 1977; Moreno, 1989; Muñoz et al., 2016; Petruzzi et al., 2006; Test, 1955). However, these studies are typically isolated to specific geographic regions or populations. A clear next step is to compare variation in these traits between color morphs in individuals collected from populations with significant differences in annual temperature and forest cover.

Second, our study focused on how abiotic factors could maintain color polymorphism, but there may be additional biotic selection pressures that favor striped individuals at cool sites with low forest cover. For example, predation pressure may vary between striped and unstriped morphs due to differences in coloration (Lotter & Scott, 1977), behavior (Moreno, 1989; Venesky & Anthony, 2007; Otaibi, Johnson, & Cosentino, In press) or local frequency (i.e., frequency‐dependent selection; Fitzpatrick, Shook, & Izally, 2009). Striped and unstriped individuals also vary in diet (Anthony et al., 2008), territorial behavior (Reiter, Anthony, & Hickerson, 2014), and disease resistance (Venesky et al., 2015). There are a host of evolutionary mechanisms that can maintain color polymorphism in animals (McLean & Stuart‐Fox, 2014), and thermal selection imposed by climate and land use likely operate with other spatially variable selection pressures to affect color polymorphism.

We did not find a relationship between *P. cinereus* morph frequencies and sampling year, which is consistent with Moore and Ouellet (2015) but in contrast to Gibbs and Karraker (2006). Gibbs and Karraker (2006) hypothesized that a negative association between proportion striped and sampling year could be due to increasing temperature associated with climate change and forest loss over time. It is possible that temporal changes in morph frequencies associated with climate warming or forest loss have been partly balanced by century‐long forest regeneration in some sectors of the study area (e.g., the northeastern United States; Thompson, Carpenter, Cogbill, & Foster, 2013). Our analyses of temporal trends in morph frequencies are also complicated by spatial biases in sampling over time (e.g., negative relationship between sampling year and annual temperature; Figure 2). Moreover, our space‐for‐time approach for inferring climate and land use effects is limited because evolutionary responses in the past do not indicate the likelihood of future evolutionary change (Fukami & Wardle, 2005; Merilä & Hendry, 2014; Urban, Richardson, & Freidenfelds, 2014). Longitudinal studies will be essential for providing direct insight into how climate and landscape change interact to shape species traits.

Our study sheds new light on the potential for evolutionary responses of species to global change. Extinction or geographic range shift is often the expectation for biotic responses to global change, but species can exhibit rapid evolutionary responses to environmental stressors manifested in morphological, physiological, or behavioral traits (Bradshaw & Holzapfel, 2001; Brady, 2012; Carroll, Hendry, Reznick, & Fox, 2007; Harris, Munshi‐South, Obergfell, & O'Neill, 2013; Réale, McAdam, Boutin, & Berteaux, 2003; Snell‐Rood & Wick, 2013). However, we generally lack knowledge of how multiple selective forces interact to shape evolution (Wade & Kalisz, 1990), including components of global change (Merilä & Hendry, 2014; Reusch & Wood, 2007). Few studies have examined interactive effects of multiple stressors on intraspecific variation. For example, ambient temperature reduces songbird productivity only in highly forested landscapes, likely due to high nest predation compared to less forested landscapes (Cox, Thompson, Reidy, & Faaborg, 2013). Our study on *P. cinereus* color morphology shows that climate and land use can have interactive effects on genetically based traits, suggesting that predictions about evolutionary responses to global change may not be straightforward. In our system, directional selection imposed by regional climate warming should cause frequency of striped individuals to decrease, but selection imposed by climate warming is likely amplified by forest loss and dampened by forest regeneration. This mediating effect of forest change is expected to be greatest in warm climates. These results suggest that evolutionary trajectories for temperature‐sensitive species may vary among populations even under consistent directional selection imposed by climate warming. Maintaining and increasing forest cover in some regions may be an effective strategy for reducing the strength of selection caused by climate change, particularly for species with constrained upper thermal limits (e.g., terrestrial ectotherms; Hoffmann, Chown, & Clusella‐Trullas, 2013).

## CONFLICT OF INTEREST

None declared.
