# Supplementary material for: Evolutionary response to global change: Climate and land use interact to shape color polymorphism in a woodland salamander
Source: Ecol Evol. 2017 Jun 12;7(14):5426–34. doi: 10.1002/ece3.3118 (PMC5528218; doi:10.1002/ece3.3118)
Supplement: Supplementary file 1 [file ECE3-7-5426-s001.pdf]

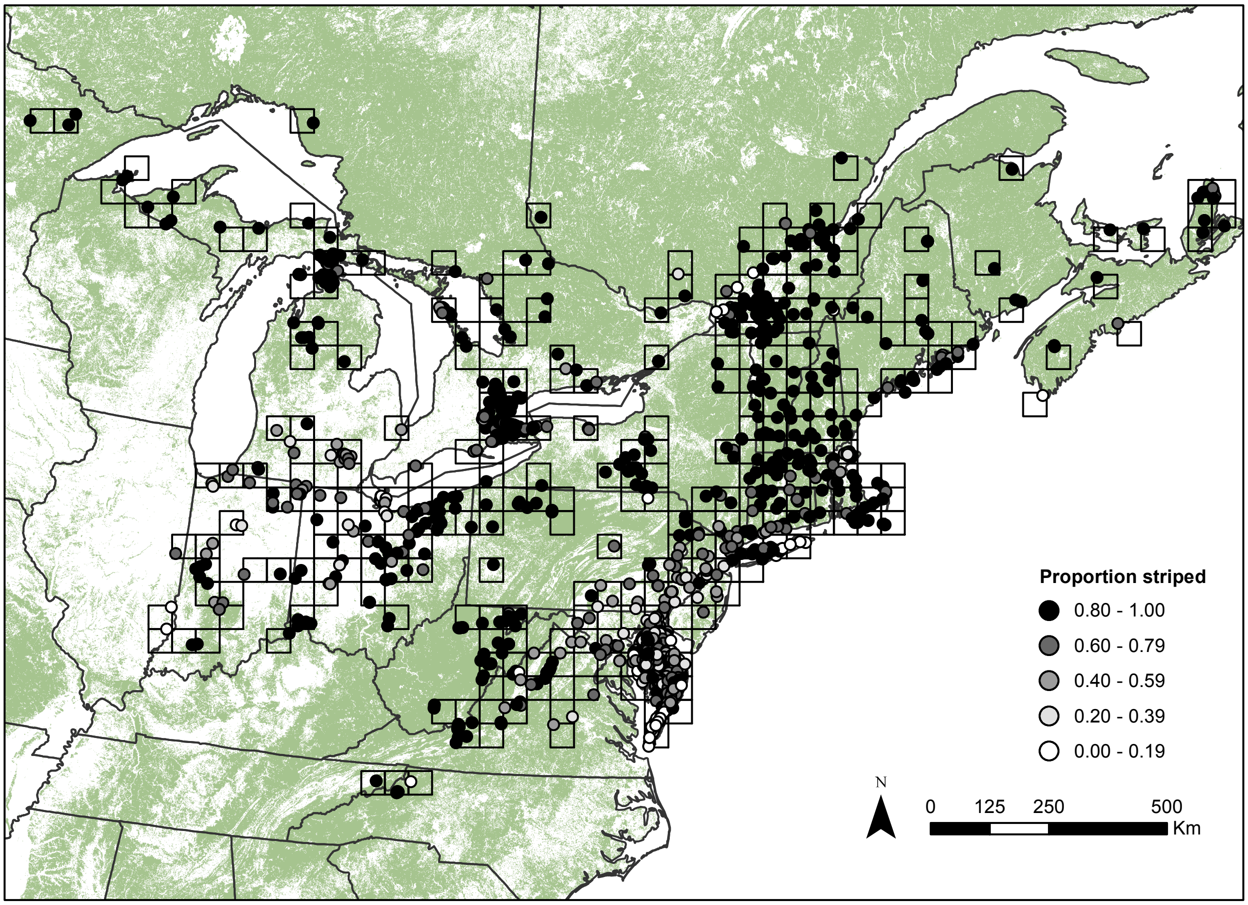

Fig. S1. Map of forest cover (green), study sites (circles), and 50x50-km landscape blocks (grid cells) for eastern red-backed salamanders (*Plethodon cinereus*) sampled from 1880 to 2015 in North America. Symbol fill for study sites represents observed proportion of striped *P. cinereus*.

Table S1. Standardized coefficients for regressions and covariances in a path analysis model of *Plethodon cinereus* morph frequencies in landscape blocks at varying spatial scales (10 x 10 km, 50 x 50 km, 100 x 100 km) and when the minimum number of individuals (*n*) at each site is 20 (*N* = 1170 sites), 30 (*N* = 848 sites), 40 (*N* = 667 sites), and 50 (*N* = 574 sites). Bold indicates significant coefficients at $P \leq 0.05$.

			Standardized coefficients			
Spatial scale	Relationship	Variables	$n \geq 20$	$n \geq 30$	$n \geq 40$	$n \geq 50$
10 x 10 km	Regressions	Striped ← Temperature	**-0.383**	**-0.358**	**-0.325**	**-0.326**
		Striped ← Forest	**0.297**	**0.312**	**0.325**	**0.305**
		Striped ← Temperature*Forest	**0.088**	**0.106**	**0.102**	0.084†
		Temperature ← Year	**-0.349**	**-0.281**	**-0.182**	**-0.136**
		Forest ← Year	**0.152**	**0.167**	**-0.152**	**0.165**
	Covariances	Temperature, Forest	-0.110	-0.079	-0.037	-0.030
50 x 50 km	Regressions	Striped ← Temperature	**-0.347**	**-0.320**	**-0.278**	**-0.280**
		Striped ← Forest	**0.335**	**0.353**	**0.362**	**0.343**
		Striped ← Temperature*Forest	**0.152**	**0.171**	**0.162**	0.122*
		Temperature ← Year	**-0.362**	**-0.299**	**-0.201**	**-0.154**
		Forest ← Year	**0.171**	**0.167**	0.120	0.122
	Covariances	Temperature, Forest	**-0.253**	**-0.226**	-0.172	-0.159
100 x 100 km	Regressions	Striped ← Temperature	**-0.350**	**-0.327**	**-0.288**	**-0.293**
		Striped ← Forest	**0.320**	**0.344**	**0.360**	**0.351**
		Striped ← Temperature*Forest	**0.162**	**0.186**	**0.176**	**0.142**
		Temperature ← Year	**-0.371**	**-0.308**	**-0.209**	-0.164
		Forest ← Year	**0.209**	**0.199**	0.146	0.127
	Covariances	Temperature, Forest	-0.197	-0.149	-0.070	-0.045

†$P = 0.075$, *$P = 0.055$

Table S2. Confidence intervals (95%) for standardized coefficients based on 1000 iteractions of a path analysis model when randomly choosing one site within each 50 x 50-km landscape block (n = 292) in each iteration.

Relationship	Variables	$Low_{95\%}$	$High_{95\%}$
Regressions	Striped ← Temperature	-0.41	-0.31
	Striped ← Forest	0.24	0.34
	Striped ← Temperature*Forest	0.12	0.23
	Temperature ← Year	-0.33	-0.24
	Forest ← Year	0.21	0.31
Covariances	Temperature, Forest	-0.33	-0.31
